# Smokeless tobacco pack-years: Non-reporting in tobacco research

**DOI:** 10.18332/tpc/215589

**Published:** 2026-02-12

**Authors:** Saravanan Sampoornam Pape Reddy, Delfin Lovelina Francis, Manish Rathi, Sukhbir Singh Chopra

**Affiliations:** 1 Army Dental Corps, Bhopal, India; 2 Department of Public Health Dentistry, Saveetha Dental College and Hospitals, Saveetha University, SIMATS, Chennai, India; 3 Army Dental Centre (Research & Referral), New Delhi, India

**Keywords:** smokeless tobacco, pack-years

**Dear Editor,**

The concept of ‘pack-years’ in smoking research is very well established to quantitatively assess the exposure. The number of cigarette packs smoked per day multiplied by the number of years of smoking gives ‘pack-years’; where a pack is considered to have 20 cigarettes. This provides a cumulative exposure and is used commonly in smoking research. However, the research on smokeless tobacco (SLT) does not report the exposure in a standardized manner. The amount of tobacco content varies significantly in various forms of SLT, including chewing tobacco, betel quid, snuff, gutka, pan masala, etc. In order to achieve standardization and comparison of SLT studies, the calculation of pack-year equivalent for SLT is necessary. To calculate the SLT pack-year, factors such as quantification of tobacco content in each SLT product (grams per sachet or quid), frequency of usage (packets per day), and multiplying the SLT usage by the number of years used can provide cumulative SLT exposure. This calculation is difficult in research due to the variable tobacco concentrations in SLT products. Nicotine absorption is affected by the type of the product, pattern of usage, individualistic habits, and duration of contact in the oral cavity^1^.

Although calculating SLT pack-years becomes difficult to assess and standardize in tobacco research, we note below a formula for SLT pack-year equivalent as follows:

*Smokeless tobacco pack-years = [Amount of tobacco used per day (g)/Standardized pack equivalent (g)] × Years of use*

or

*Smokeless tobacco pack-years = (Daily nicotine intake/20 mg) × Number of years used*

When only mass data are available, a mass-based formula could be used:

*SLT pack-years (mass-based) = [Daily SLT mass (g)/M_ref (g)] × Years of use*

where M_ref is a study-justified ‘reference mass equivalent’.

A formula based on nicotine-yield could also be used:

*SLT pack-years (nicotine-based) = [Daily nicotine intake (mg)/D_ref (mg)] × Years of use*

where D_ref is a study-justified ‘reference nicotine dose’, selected and justified based on product-specific nicotine yield/absorption.

In the above formulae, D_ref and M_ref are not universal constants. Due to the diversity of products on the SLT market, not only in nicotine levels but also in size of product, placement, placement time, and in user behavior, a single fixed value (e.g. 20 mg) cannot be used to set an SLT threshold. Investigators should benchmark and justify D_ref (and/or M_ref) based on product-specific data (e.g. lab-derived nicotine yield or validated literature estimates) and transparently report these values to enable comparisons. It should be noted that, in contrast to cigarettes, SLT products are extremely heterogeneous in their type and volume of use. This variation is an obstacle for a single measure that can be used universally. Therefore, our proposed formulae may be considered as a concept or an outline for standardization.

Future studies should seek to quantify grams of content, product-specific nicotine yield, and frequency-of-use-based equivalents, enabling further validation and generalization. In this sense, the standardized pack equivalent, as a concept, does not represent a universal constant but is merely a researcher-chosen reference unit to homogenize reporting. This may be a reference mass (M_ref in g) or a reference nicotine dose (D_ref in mg) if known. M_ref or D_ref need to be defined using local product information or laboratory analysis, clearly justified, and reported in a way that enables replication and comparability. In such calculations, the amount of SLT used actually is the total mass of SLT consumed on daily basis.

Hence, the concept of standardized pack-equivalent is a hypothetical value which is to be used to standardize exposure to SLT. Thus, the proposed formulae provide a basis for the quantification of SLT research; the inherent problem lies in the lack of information on ‘standardized pack-equivalent’, which warrants further research to assess the SLT exposure^2^.

Individual variations in the frequency of SLT intake at different timings make it difficult to utilize standardized metrics^3,4^. In addition, most research on SLT report only the self-reported data from participants, thereby introducing higher chances of recall bias^5^.

Most surveillance systems and many epidemiological studies differentiate between lifetime (ever) use from current use for SLT, typically defining current use as having used chewing tobacco, snuff, dip, snus, or dissolvable products ‘every day’ or ‘some days’ among those who have used at least once in their lifetime. Although this method standardizes the reporting of prevalence, it does not measure total lifetime exposure.

The WHO Global Adult Tobacco Survey (GATS) furnished the standardized indicators for tobacco use, including SLT in adults across countries, with yet more emphasis on status (ever/current) and less on cumulative dose. This concept is a limitation for the dose–response analyses and cross-product comparison in SLT studies^6^.

In summary, while the health hazards of SLT are well known, the lack of a common measurement of exposure has hampered the comparability among studies. The suggested ‘SLT pack-year equivalent’ is one possible approach to fill this gap with a long-term view of facilitating better epidemiological evidence and more informed public health.

## Data Availability

Data sharing is not applicable to this article as no new data were created.
